# The effect of cycloplegia on corneal power and diameter measured by an auto-kerato-refractometer in presbyopic patients with type 2 diabetes mellitus: a post-hoc analysis of a randomized clinical trial

**DOI:** 10.1186/s12886-026-05351-1

**Published:** 2026-09-18

**Authors:** Navid Elmi Sadr, Leila Ghiasian, Yasaman Hadi, Ramyar Hariri

**Affiliations:** 1 Clinical Research Development Unit, Kowsar Educational, Research and Therapeutic Hospital, Semnan University of Medical Sciences, Semnan, Iran; 2 Department of Ophthalmology, School of Medicine, Semnan University of Medical Sciences, Semnan, Iran; 3 Eye Research Center, Eye Department, The Five Senses Health Institute, School of Medicine, Iran University of Medical Sciences, Tehran, Iran; 4 Kowsar Semnan Research and Medical Training Center, Semnan, 3514799442 Iran

**Keywords:** Cornea, Cycloplegia, Diabetes, Keratometry, Tropicamide, WTW

## Abstract

**Background:**

To assess the effect of cycloplegia on keratometry and horizontal white to white (WTW) measurements in presbyopic patients with type 2 diabetes.

**Methods:**

The post-hoc analysis included 88 eyes from 88 patients with type 2 diabetes aged >40 years. Flat keratometry (Kf), steep keratometry (Ks), mean keratometry (Km), corneal astigmatism (CA), and WTW were measured using the RC-5000 auto kerato-refractometer (Tomey Inc., Nagoya, Japan), before and thirty minutes after the instillation of two drops (administered five minutes apart) of either 0.5% (46 patients) or 1% tropicamide (42 patients).

**Results:**

The mean changes in Kf, Ks, Km, CA, and WTW were − 0.01 ± 0.26 D, -0.05 ± 0.23 D, -0.03 ± 0.19 D, -0.04 ± 0.31 D, and 0.05 ± 0.24 mm, respectively, in the 0.5% tropicamide group. These values were − 0.07 ± 0.28 D, 0.01 ± 0.23 D, -0.03 ± 0.21 D, 0.08 ± 0.29 D, and 0.04 ± 0.15 mm, respectively, in the 1% tropicamide group. The changes following cycloplegia were not statistically significant (P ˃ 0.05).

**Conclusions:**

Post-cycloplegia keratometry and WTW measurements were comparable to pre-cycloplegia values in presbyopic patients with type 2 diabetes, suggesting that these measurements can be reliably obtained after cycloplegia.

**Trial registration:**

IRCT.ir, registration number: IRCT20200829048553N1, 2021-05-31. ClinicalTrials.gov, ID: NCT04932213, 2021-06-21.

## Background

keratometry measurements are essential for calculating intraocular lens (IOL) power and are a fundamental aspect of the preoperative assessment for cataract surgery [1]. The accuracy of these measurements is particularly important for diabetic patients, as they are more susceptible to developing cataracts at an earlier age compared to the general population due to metabolic changes associated with diabetes, which accelerate lens opacification [2]. The integration of optical biometers in anterior segment measurements, along with the adoption of fourth-generation and newer-generation IOL power formulas, has significantly enhanced the refractive outcomes of cataract surgery [1]. However, in developing countries, the prevalence of diabetes and associated cataracts is significant, yet access to advanced optical biometers is often limited. As a result, ophthalmologists often use auto-kerato-refractometers and A-scan ultrasonography as cost-effective alternatives to obtain measurements needed for applying third-generation IOL power formulas. These devices provide practical and affordable solutions in resource-limited settings, ensuring patients receive accurate assessments essential for optimal surgical outcomes. Additionally, measuring refraction, corneal power, and corneal diameter plays a crucial role in prescribing contact lenses through empirical fitting [3, 4]. In diabetic patients, these measurements must exhibit exceptional accuracy because of the specific challenges associated with contact lens suitability in this population [5, 6]. Corneal topography and auto-kerato-refractometers are commonly used for this purpose. However, in areas with limited resources, auto-kerato-refractometers may be the only available instrument in ophthalmology and optometry clinics.

Performing a comprehensive dilated fundus examination to screen for diabetic retinopathy and macular edema is essential before considering cataract surgery or prescribing contact lenses. The presence and severity of retinopathy or maculopathy are critical factors that can affect treatment plans. Therefore, in a busy clinical setting, this evaluation might be conducted before taking corneal measurements during the same visit to save time and reduce costs. Several studies have explored the impact of cycloplegia and pupillary dilation on corneal parameters in healthy individuals [7–12]. However, the results have been contradictory, and there is a lack of research specifically addressing this issue in diabetic patients [13]. Accordingly, the objective of this study was to assess the keratometry and WTW values obtained from the RC-5000 auto-kerato-refractometer (Tomey Inc., Nagoya, Japan) before and after cycloplegia induced by two different concentrations of tropicamide (0.5% or 1%) in a group of presbyopic patients with type 2 diabetes mellitus.

## Methods

### Study design

The post-hoc analysis included 88 eyes from 88 patients aged ˃ 40 years diagnosed with type 2 diabetes mellitus who participated in a clinical trial registered at ClinicalTrials.gov with the identifier NCT04932213. The clinical trial was conducted at the Ophthalmology Clinic of Kowsar Hospital (Semnan University of Medical Sciences, Semnan, Iran) from July 7, 2021 to November 7, 2021 [13, 14]. Individuals with proliferative diabetic retinopathy, pterygium, corneal dystrophies, corneal ectasia, severe nuclear or cortical cataracts, anisocoria, iris disorders, iris neovascularization, elevated intraocular pressure, narrow angles, glaucoma, use of miotics or mydriatics, and previous refractive or cataract surgery were excluded. All participants provided written informed consent. The study followed the principles outlined in the Declaration of Helsinki and was approved by the Institutional Ethics Committee.

### Study conduct

An ophthalmologist (NES) performed a comprehensive ophthalmological examination of all patients. The measurements were performed by a skilled operator using the Tomey RC-5000 auto-kerato-refractometer. The measured parameters included the sphere (SPH), negative cylinder (CYL), spherical equivalent (SE) calculated as (SPH + CYL/2), flat keratometry (Kf), steep keratometry (Ks), mean keratometry (Km) calculated as ((Kf + Ks) / 2), corneal astigmatism (CA) calculated as (Ks – Kf), pupillary diameter (PD), and horizontal white to white distance (WTW). The keratometry measurements were taken within a 3.00 mm diameter area of the central cornea. The average of three consecutive automated keratometry measurements, without head repositioning between measurements, was used for the analysis. The pupillary diameter and WTW were determined as the distance between two vertical lines manually positioned at the nasal and temporal edges of the pupil and corneal limbus in a single image, respectively. The nurse administered either 0.5% or 1% tropicamide to one eye of each patient, with one drop administered every 5 min for a total of two administrations. It is important to note that the study participants, the operator, and the ophthalmologist were blinded to the specific medication used for each patient. Thirty minutes later, the measurements were repeated. The primary outcome measures focused on assessing the changes in Kf, Ks, Km, WTW, and PD from the baseline. Before each study session, the auto-kerato-refractometer was calibrated using the manufacturer’s model eye to ensure precision and reproducibility. To minimize the effect of diurnal variation, all assessments were carried out between 10 am and 1 pm.

In addition, the contralateral eye of each patient underwent three consecutive automated keratometry measurements. Thirty minutes later, a second set of three consecutive measurements was performed. The average values of Ks, Kf, and Km for each set of measurements were compared to evaluate device repeatability.

### Statistical analysis

Statistical analyses were performed using SPSS software for Windows version 25 (SPSS Inc., Chicago, IL, U.S.). Statistical significance was set at p-values below 0.05. Keratometry and WTW values were compared before and after cycloplegia using the paired samples t-test and Wilcoxon signed-rank test, respectively. Agreement between pre- and post-dilation measurements was evaluated using Bland–Altman analysis. The 95% limits of agreement (LoA) were calculated as the mean difference ± 1.96 × the standard deviation of the paired differences. To evaluate the repeatability, the paired samples t-test, intraclass correlation coefficients (ICC), within-subject standard deviation (Sw), repeatability coefficient of 2.77 × Sw, and coefficient of variation were employed [15, 16]. The Pearson or Spearman correlation coefficient was employed to evaluate the relationship between post-cycloplegia changes in corneal measurements and baseline characteristics.

## Results

Forty-six patients with a mean age of 57.61 ± 7.13 years (range: 42 to 72) and 42 patients with a mean age of 56.71 ± 8.88 years (range: 42 to 76) were included in the 0.5% tropicamide group and 1% tropicamide group, respectively. Table 1 shows the demographic and clinical characteristics of the patients. The Pearson correlation analysis indicated that there was no significant correlation between the post-cycloplegia changes in Kf, Ks, Km, and CA with factors including age, sex, duration of diabetes, diabetic retinopathy status, and tropicamide concentration (*P* > 0.05 for all). Nevertheless, a weak positive correlation was found between the change in WTW and sex, as indicated by Spearman’s Rho coefficient of 0.26 (*P* = 0.01).

**Table 1:** The demographic and clinical characteristics of the patients

Variables	0.5% Tropicamide	1% Tropicamide
Sex		
Male	20	24
Female	26	18
Eye laterality		
Right	26	26
Left	20	16
Diabetic retinopathy		
None	27	25
Mild NPDR	9	6
Moderate NPDR	6	8
Severe NPDR	4	3
CSME	0	1
Diabetes duration (years since diagnosis)		
≤ 10	35	29
˃ 10	11	13

NPDR, none-proliferative diabetic retinopathy defined by the Diabetic Retinopathy Study (DRS); CSME, clinically significant macular edema defined by the Early Treatment Diabetic Retinopathy Study (ETDRS)

### The 0.5% tropicamide group

The mean change in Kf, Ks, Km, CA, and WTW was found to be -0.01 ± 0.26 D, -0.05 ± 0.23 D, -0.03 ± 0.19 D, -0.04 ± 0.31 D, and 0.05 ± 0.24 mm, respectively, as indicated in Table 2. There were no statistically significant differences in keratometry or WTW values before and after the administration of 0.5% tropicamide (P ˃ 0.05). However, post-dilation PD exhibited a significant increase with a mean of 2.01 ± 0.68 mm (P ˂ 0.001). In addition, SPH and SE increased significantly (*P* ≤ 0.001).

**Table 2:** The mean ± SD (95% CI) values of corneal parameters and PD, before and after 0.5% tropicamide administration

Variables	Pre-dilation	Post-dilation	change	*p*-value
SPH (D)	0.53 ± 0.19 (0.15, 0.90)	0.78 ± 0.18 (0.43, 1.14)	0.25 ± 0.44	˂ 0.001*
CYL (D)	-0.97 ± 0.12 (-1.21, -0.72)	-0.98 ± 0.14 (-1.27, -0.69)	-0.01 ± 0.58	0.85*
SE (D)	0.04 ± 0.20 (-0.37, 0.46)	0.29 ± 0.20 (-0.11, 0.69)	0.25 ± 0.49	0.001*
Kf (D)	44.17 ± 1.17 (43.83, 44.23)	44.16 ± 1.25 (43.79, 44.53)	-0.01 ± 0.26	0.77*
Ks (D)	45.00 ± 1.25 (45.37, 44.63)	44.94 ± 1.28 (44.56, 45.32)	-0.05 ± 0.23	0.12*
Km (D)	44.58 ± 1.16 (44.24, 44.93)	44.55 ± 1.21 (44.19, 44.91)	-0.03 ± 0.19	0.24*
CA (D)	0.82 ± 0.74 (0.61, 1.04)	0.78 ± 0.76 (0.56, 1.01)	-0.04 ± 0.31	0.34*
PD (mm)	4.40 ± 0.86 (4.14, 4.65)	6.41 ± 0.88 (6.15, 6.67)	2.01 ± 0.68	˂ 0.001*
WTW (mm)	12.03 ± 0.45 (11.89, 12.16)	12.07 ± 0.37 (11.96, 12.18)	0.05 ± 0.24	0.18**

SPH, sphere; CYL, cylinder; SE, spherical equivalent; Kf, flat keratometry; Ks, steep keratometry; Km, mean keratometry; CA, corneal astigmatism; PD, pupillary diameter; WTW, white to white distance. * Paired-Samples T test, ** Wilcoxon signed-rank test

### The 1% tropicamide group

Table 3 presents the mean changes in Kf, Ks, Km, CA, and WTW, which were determined to be -0.07 ± 0.28 D, 0.01 ± 0.23 D, -0.03 ± 0.21 D, 0.08 ± 0.29 D, and 0.04 ± 0.15 mm, respectively. There were no statistically significant differences in keratometry or WTW values before and after 1% tropicamide administration (P ˃ 0.05). However, post-dilation PD increased significantly with a mean increase of 2.15 ± 0.620 mm (P ˂ 0.001). In addition, SPH and SE increased significantly (*P* ≤ 0.001).

**Table 3:** The mean ± SD (95% CI) values of corneal parameters and PD, before and after 1% tropicamide administration

Variables	Pre-dilation	Post-dilation	change	*p*-value
SPH (D)	0.14 ± 0.25 (-0.37, 0.64)	0.40 ± 0.24 (-0.10, 0.89)	0.26 ± 0.41	˂ 0.001*
CYL (D)	-0.91 ± 0.10 (-1.11, -0.70)	-0.95 ± 0.11 (-1.17, -0.72)	-0.03 ± 0.31	0.46*
SE (D)	-0.32 ± 0.26 (-0.84, 0.20)	-0.07 ± 0.25 (-0.57, 0.42)	0.24 ± 0.42	0.001*
Kf (D)	43.84 ± 1.46 (43.39, 44.30)	43.77 ± 1.45 (43.32, 44.22)	-0.07 ± 0.28	0.11*
Ks (D)	44.61 ± 1.56 (44.12, 45.09)	44.62 ± 1.49 (44.15, 45.08)	0.01 ± 0.23	0.74*
Km (D)	44.21 ± 1.49 (43.75, 44.68)	44.18 ± 1.43 (43.73, 44.62)	-0.03 ± 0.21	0.28*
CA (D)	0.76 ± 0.51 (0.60, 0.92)	0.84 ± 0.56 (0.67, 1.02)	0.08 ± 0.29	0.07*
PD (mm)	4.59 ± 0.78 (4.34, 4.83)	6.74 ± 0.78 (6.50, 6.98)	2.15 ± 0.620	˂ 0.001*
WTW (mm)	12.14 ± 0.51 (11.98, 12.30)	12.18 ± 0.50 (12.02, 12.33)	0.04 ± 0.15	0.11**

SPH, sphere; CYL, cylinder; SE, spherical equivalent; Kf, flat keratometry; Ks, steep keratometry; Km, mean keratometry; CA, corneal astigmatism; PD, pupillary diameter; WTW, white to white distance. * Paired-Samples T test, ** Wilcoxon signed-rank test

### Bland–Altman analysis

**Fig. 1:**
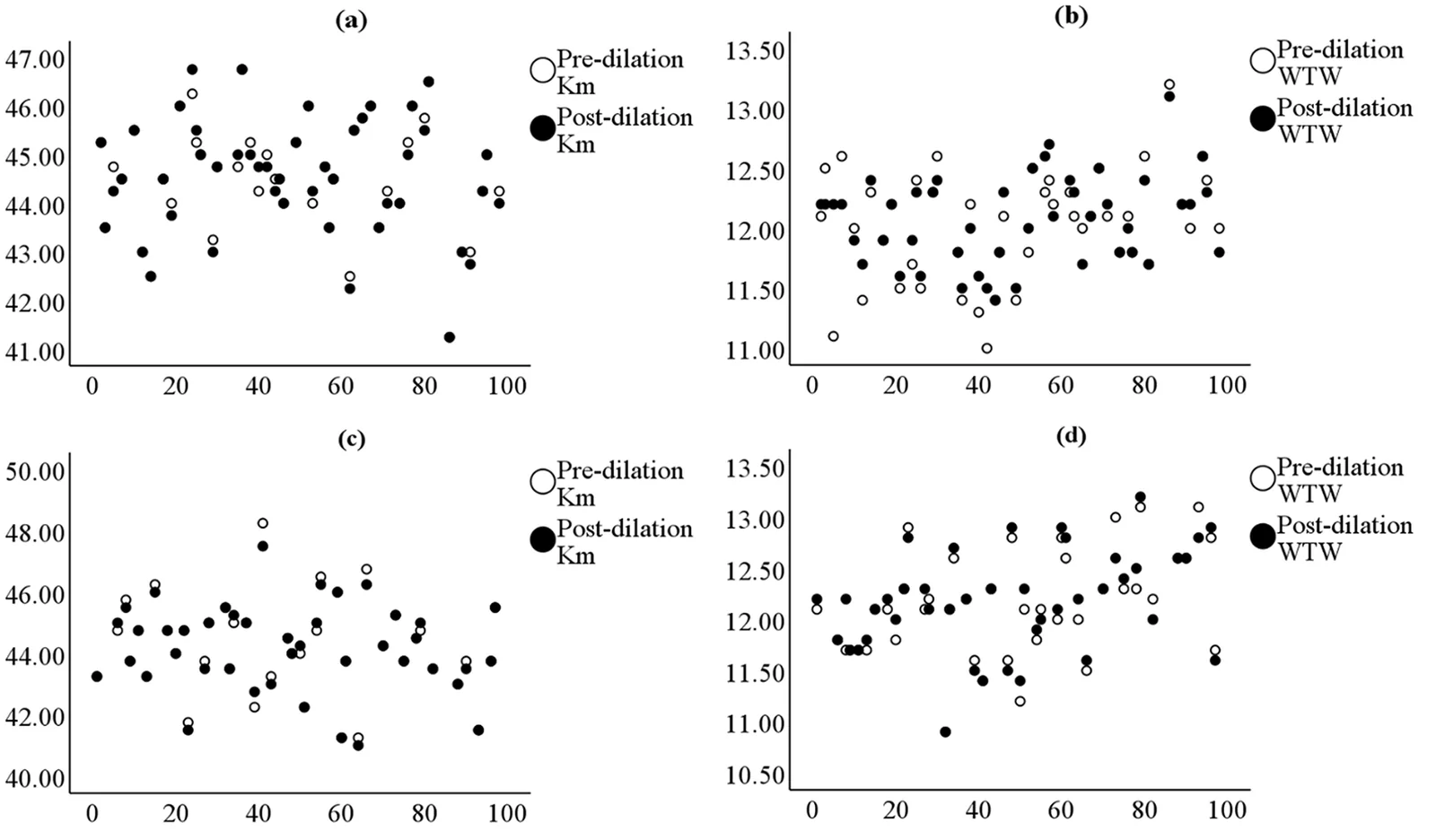
Distribution of pre- and post-dilation Km and WTW measurements in the 0.5% and 1% tropicamide groups. (**a**) Km and, (**b**) WTW measurement distributions before and after dilation in the 0.5% tropicamide group;, (**C**) Km and, (**D**) WTW measurement distributions before and after dilation in the 1% tropicamide group. The x-axis represents the participant number. Km, mean keratometry; WTW, white-to-white distance

**Fig. 2:**
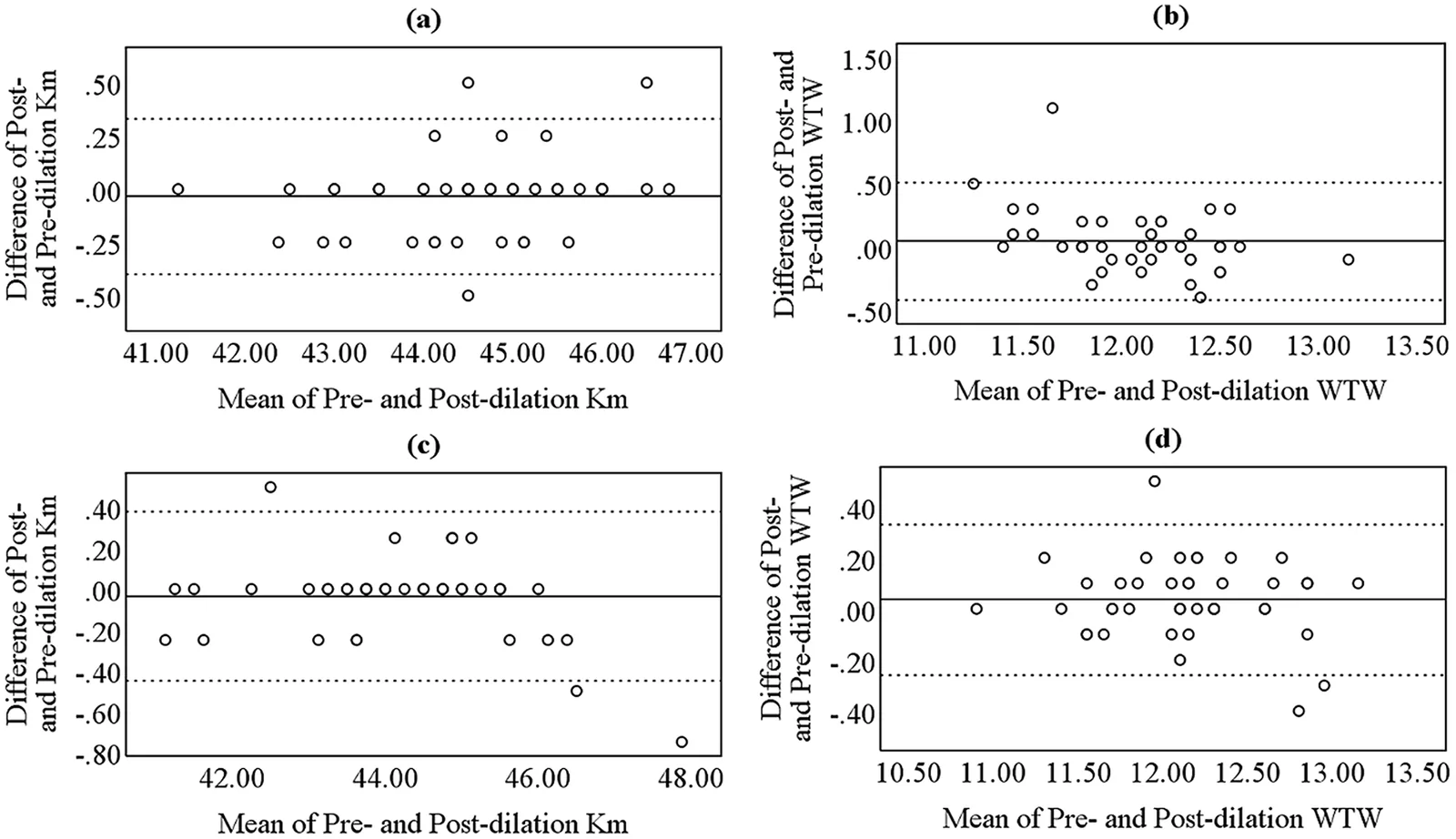
Bland-Altman plots for Km and WTW. Bland-Altman plots for pre- and post-dilation (**a**) Km and (**b**) WTW in the 0.5% tropicamide group. Bland-Altman plots for pre- and post-dilation (**c**) Km and (**d**) WTW in the 1% tropicamide group. The solid line represents the mean difference, and the upper and lower dashed lines represent the 95% limits of agreement (mean difference ± 1.96 SD). Km, mean keratometry; WTW, white to white distance; SD, standard deviation

Figure 1 illustrates the distribution of pre- and post-dilation Km and WTW measurements in the 0.5% and 1% tropicamide groups. Figure 2 presents the corresponding Bland–Altman plots, demonstrating good agreement between pre- and post-dilation measurements. The 95% LoA for Km ranged from − 0.40 to 0.33 D in the 0.5% tropicamide group and from − 0.45 to 0.38 D in the 1% tropicamide group. For WTW, the 95% LoA ranged from − 0.42 to 0.51 mm in the 0.5% tropicamide group and from − 0.26 to 0.33 mm in the 1% tropicamide group.

### Between-group comparison

Table 4 presents the mean changes in SPH, CYL, SE, Kf, Ks, Km, CA, PD, and WTW following cycloplegia in the 0.5% and 1% tropicamide groups. No statistically significant differences were observed between the two groups in the mean changes of any measured parameter (all *P* > 0.05).

**Table 4:** Mean ± SD changes in refraction, corneal parameters, and PD following administration of 0.5% and 1% tropicamide

Variables	0.5% Tropicamide	1% Tropicamide	*p*-value*
SPH (D)	0.25 ± 0.44	0.26 ± 0.41	0.94
CYL (D)	-0.01 ± 0.58	-0.03 ± 0.31	0.84
SE (D)	0.25 ± 0.49	0.24 ± 0.42	0.97
Kf (D)	-0.01 ± 0.26	-0.07 ± 0.28	0.30
Ks (D)	-0.05 ± 0.23	0.01 ± 0.23	0.19
Km (D)	-0.03 ± 0.19	-0.03 ± 0.21	0.94
CA (D)	-0.04 ± 0.31	0.08 ± 0.29	0.053
PD (mm)	2.01 ± 0.68	2.15 ± 0.620	0.30
WTW (mm)	0.05 ± 0.24	0.04 ± 0.15	0.82

SPH, sphere; CYL, cylinder; SE, spherical equivalent; Kf, flat keratometry; Ks, steep keratometry; Km, mean keratometry; CA, corneal astigmatism; PD, pupillary diameter; WTW, white to white distance. * Independent-Samples T test

### Repeatability

The mean ± SD (95% CI) values for Kf, Ks, and Km were 43.87 ± 1.43 D (43.57, 44.18), 44.77 ± 1.34 D (44.49, 45.05), and 44.29 ± 1.33 D (44.01, 44.57), respectively, for the first set of measurements. For the second set of measurements, the mean ± SD (95% CI) values for Kf, Ks, and Km were 43.98 ± 1.35 D (43.69, 44.26), 44.79 ± 1.38 D (44.50, 45.08), and 44.37 ± 1.32 D (44.09, 44.65), respectively. There was no significant difference between the two sets of measurements, regarding Kf, Ks, and Km (P ˃ 0.05). Furthermore, the two sets of measurements exhibited excellent correlation, with an ICC of at least 0.977 (*P* < 0.001) for all variables, as presented in Table 5.

**Table 5:** Repeatability of the Tomey RC-5000 auto-kerato-refractometer

Variables	Mean ± SD	Sw	2.77 × Sw	CoV%	ICC (95% CI)
Kf (D)	43.87 ± 1.43	0.22	0.61	0.50	0.988 (0.981, 0.992)
Ks (D)	44.76 ± 1.32	0.29	0.80	0.65	0.977 (0.965, 0.985)
Km (D)	44.28 ± 1.33	0.15	0.41	0.34	0.994 (0.991, 0.996)

Kf, flat keratometry; Ks, steep keratometry; Km, mean keratometry; Sw, within-subject standard deviation; CoV, coefficient of variation; ICC, intraclass correlation coefficient (two-way mixed effects, absolute agreement, single measures)

## Discussion

Diabetes adversely affects the anterior segment of the eye, leading to corneal neuropathy, dry eye disease, delayed corneal healing, refractive instability, and early cataract [2, 6]. Thus, patients with diabetes are prone to postoperative complications, and accurate measurement of corneal parameters is critical to avoid undesired outcomes following cataract surgery and during contact lens wear. We assessed the changes in corneal power and diameter following administration of 0.5% and 1% tropicamide in a group of presbyopic patients with type 2 diabetes using an auto-kerato-refractometer. The RC-5000 is a fully automated kerato-refractometer, which provides measurements of corneal curvature radius within a range of 5.00 mm to 11.00 mm. The corneal refractive powers are then computed by employing a corneal refractive index of *n* = 1.3375 using the Gaussian Eq. 337.5 / (radius of curvature in mm) [16, 17]. In our study, the RC-5000 showed good repeatability for the measurements of Kf, Ks, and Km, which is in line with a previous study [16].

While some studies have reported no remarkable change in keratometry following cycloplegia [7–9, 13], others have shown significant corneal flattening [10–12]. Elmi Sadr et al. [13] showed that the use of 0.5% or 1% tropicamide for cycloplegia did not affect the anterior and posterior corneal curvatures as measured by Pentacam in diabetic patients. In contrast, a study involving healthy individuals with an average age of 31.1 ± 5.6 years found that cycloplegia induced by tropicamide/phenylephrine drops led to flattening of both the anterior and posterior corneal surfaces [12]. Using IOLMaster, Cheng et al. [11] found that flat and steep keratometry values decreased after cycloplegia with tropicamide in children with a mean age of 9.1 ± 2.8 years; They explained that ciliary muscle paralysis induced by the cycloplegic drug reduces the force exerted on the scleral spur which results in corneal flattening. Our study demonstrated that cycloplegia with tropicamide had no effect on keratometry. This lack of effect could be attributed to the fact that our patients belonged to the presbyopic age category (˃40 years). It can be argued that the difference in the forces exerted on the scleral spur before and after cycloplegia might not substantially alter corneal curvature among individuals with presbyopia due to age-related changes in the ciliary muscle [18]. Another potential mechanism that could contribute to this phenomenon in patients with diabetes is the effect of diabetic autonomic neuropathy, which weakens the ciliary muscle and diminishes its response to cycloplegic medications [19].

The current study found no change in WTW following cycloplegia, which is in line with previous studies [7, 20]. In contrast, Elmi Sadr et al. [13] showed that WTW increased following cycloplegia induced by administering either 0.5% or 1% tropicamide, as assessed using Pentacam HR in diabetic patients. Corneal flattening results from the reduced force on the scleral spur following cycloplegia [11]. It is hypothesized that this occurrence could lead to a genuine widening of WTW. However, since there was no change in corneal power in the study, the widening of WTW observed in Pentacam measurements may not be a true anatomical widening but rather a result of modifications in image analysis by the internal software, which constructs a 3-D image of the anterior segment by combining multiple images. The Pentacam HR uses a rotating Scheimpflug camera to acquire multiple images of the anterior segment and reconstruct a three-dimensional image, with WTW subsequently determined through image analysis [21]. Consequently, the bunching of the iris towards the angle following cycloplegia could affect the determination of landmark boundaries. This issue has been previously addressed in IOL Master and Lenstar [22]. In contrast, the boundaries in the RC-5000 single image were manually determined by the operator and were not influenced by such image processing.

The current study has several limitations. First, the conclusions were drawn from a relatively small sample size that was assessed using a retrospective analysis, which may limit the internal and external validity of the results. Second, our study was restricted to presbyopic patients with type 2 diabetes; therefore, the findings are not directly applicable to younger patients with diabetes or non-diabetic populations. Future studies should investigate the effects of cycloplegia on corneal parameters in broader age and disease groups. Third, our study did not include individuals with corneal ectasia. Thus, the findings of this study may not be applicable to this specific group. In addition, patients with PDR were excluded; therefore, the present findings may not be generalizable to patients with PDR. Lastly, our study was limited to intraobserver repeatability, prompting further exploration of the impact of different operators on measurement variability.

## Conclusions

In conclusion, this study showed that keratometry and WTW measurements acquired using an auto-kerato-refractometer, which may provide a practical and affordable option in resource-limited settings, did not differ significantly before and after cycloplegia with either 0.5% or 1% tropicamide in presbyopic patients with type 2 diabetes. These findings suggest that, in busy clinical settings, these corneal parameters may be measured either before or after pharmacological pupillary dilation during the same visit, potentially reducing the need for a separate visit for corneal measurements. However, because this study did not directly evaluate IOL power calculations or contact lens fitting outcomes, future studies assessing the direct impact of cycloplegia on IOL power calculation, postoperative refractive outcomes, and contact lens fitting in this population are warranted.

## Data Availability

The datasets analyzed during the current study are available in the Harvard Dataverse repository, https://doi.org/10.7910/DVN/ODSLLZ.

## Abbreviations

SPH: Sphere
CYL: Cylinder
SE: Spherical equivalent
WTW: Horizontal white to white distance
PD: Pupillary diameter
Ks: Steep keratometry
Kf: Flat keratometry
Km: Mean keratometry
CA: Corneal astigmatism
